# Supplementary material for: Spheroplasts preparation boosts the catalytic potential of a squalene-hopene cyclase
Source: Nat Commun. 2022 Oct 21;13:6269. doi: 10.1038/s41467-022-34030-0 (PMC9586974; doi:10.1038/s41467-022-34030-0)
Supplement: Supplementary file 1 — Supplementary information [file 41467_2022_34030_MOESM1_ESM.pdf]

Supplementary Information

Spheroplasts preparation boosts the catalytic potential of a squalene-hopene cyclase

Ana I. Benítez-Mateos[1], Andreas Schneider[2], Eimear Hegarty[1], Bernhard Hauer[2]*, and Francesca Paradisi[1,]*

[1] Department of Chemistry, Biochemistry and Pharmaceutical Sciences. University of Bern Freiestrasse 3, 3012 Bern (Switzerland)

[2] Institute of Biochemistry and Technical Biochemistry, University of Stuttgart Allmandring 31, 70569 Stuttgart-Vaihingen (Germany)

*Correspondence: francesca.paradisi@unibe.ch; bernhard.hauer@itb.uni-stuttgart.de

Content

1. **Supplementary Discussion** 2
2. **Supplementary Methods** 23
3. **Supplementary References** 26

1. Supplementary Discussion

Supplementary Table 1. Batch biotransformations with entrapped whole cells into alginate beads using 1*E/Z* as substrate. 200 mg of entrapped cells were added to 1 mL geranyl acetone **1*E/Z***, 1% DMSO, 0.2% SDS, and 20 mM citric acid buffer at pH 6.0 and incubated at 30ºC. A) Different substrates concentrations were used for 24 h biotranformations. B) 10 mM of **1*E/Z*** was used for the biotransformations at different incubation times. Reactions were performed in technical duplicates with a standard deviation <10%.

A

Substrate concentration	Molar conversion (%)
10 mM	7
5 mM	4

B

Time (h)	Molar conversion (%)
24	7.2
48	7.1
72	7.3

Supplementary Table 2. Continuous flow biotransformations with entrapped whole cells into alginate beads using 1*E/Z* as substrate. Substrate solution: 10 mM geranyl acetone **1*E/Z***, 1% DMSO, 0.2% SDS, and 20 mM citric acid buffer at pH 6.0. Reactor volume: 2.5 mL. The conversion was calculated as the average of at least 3 column volumes with a standard deviation <1%.

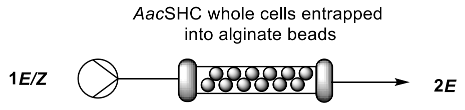

Residence time (min)	Flow-rate (µL/min)	Temperature (ºC)	Molar conversion (%)
30	80	30	<1
60	40	30	2.5
		50	<1

After the biotransformations with immobilized cells on solid supports, a decrease of the substrate **1*E/Z*** was detected while no product was found, especially when using the methacrylate microbeads which present a higher hydrophobicity (Supplementary Fig. 1B). Therefore, we imagined that the substrate **1*E/Z*** could either get stuck to the agarose/methacrylate support or trapped inside the cells. To decipher the spatial location of the substrate, we firstly extracted both substrate and product using two non-polar solvents such as cyclohexane and toluene. Only traces of product were detected in both cases, whereas the detection of substrate remained at 75±9% from the agarose microbeads and only 36±5% from the methacrylate microbeads (Supplementary Fig. 2A). Secondly, the substrate was incubated with agarose microbeads (without immobilized cells), methacrylate microbeads (without immobilized cells), and whole cells. This experiment revealed that most of the substrate (80%) is trapped inside the cells, although the stickiness to the agarose/methacrylate microbeads is a hurdle as well (Supplementary Fig. 2B).

A

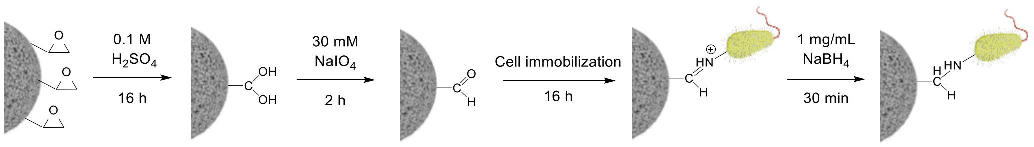

B

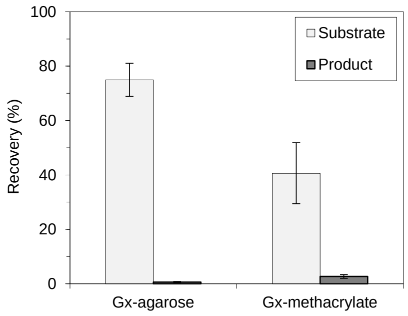

C

Temperature (ºC)	Molar conversión (%)
25	3.1
30	3.2
37	3.4

Supplementary Fig. 1. Immobilized whole cells on premade agarose (6BCL) and methacrylate (EP403/S) supports. A) Scheme of the activation of the microbeads with glyoxyl (Gx) groups and the immobilization of whole cells. The glyoxyl groups could interact with the lysines of the proteins on the cell membrane stablishing covalent bonds. B) Batch biotransformations with the immobilized whole cells. 200 mg of microbeads with immobilized cells (36 $mg_{cells}/g_{support}$) were added to 1 mL of 10 mM geranyl acetone **1*E/Z***, 1% DMSO, 0.2% SDS, and 20 mM citric acid buffer at pH 6.0. The reactions were incubated at 30 ºC for 24 h. The recovery (%) of substrate and product were calculated by using standard curves of the substrate and product. The bar chart represents the average of technical duplicates. Data are presented as mean values +/- SD. C) The biotransformations were incubated at different temperatures for 24 h. Reactions were performed in technical duplicates.

A

Support	Extraction solvent	Substrate recovery (%)	Product recovery (%)
Gx-agarose	Cyclohexane	68	<2
	Toluene	81	<2
Gx-methacrylate	Cyclohexane	32	<2
	Toluene	39	<2

B

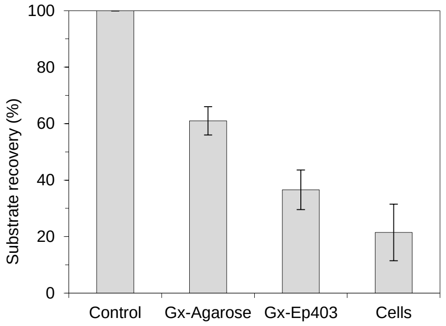

Supplementary Fig. 2. Extraction tests of substrate 1*E/Z* and product. A) Testing the extraction efficiency of non-polar solvents (1:1) for **1*E/Z*** and **2*E***. Biotransformations were performed with 200 mg of microbeads with immobilized cells (36 $mg_{cells}/g_{support}$) in 1 mL of 10 mM **1*E/Z***, 1% DMSO, 0.2% SDS, and 20 mM citric acid buffer at pH 6.0 at 30 ºC for 24 h. B) The supports (without immobilized cells), and the cells were incubated with 1 mL of 10 mM **1*E/Z***, 1% DMSO, 0.2% SDS, and 20 mM citric acid buffer at pH 6.0 at 30 ºC for 24 h. The (%) recovery of substrate was calculated in comparison with the recovery from the substrate solution (control). The control refers to a solution of 10 mM **1*E/Z***, 1% DMSO, 0.2% SDS, and 20 mM citric acid buffer at pH 6.0 treated with the same conditions as the samples. The control does not contain any support nor cells. The bar chart represents the average of technical duplicates. Data are presented as mean values +/- SD.

The differences on the substrate extraction could be attributed to cell permeabilization effects by the pH (Supplementary Fig. 3). It has been reported that buffer at more basic pH can increase the outer membrane permeability.[1]

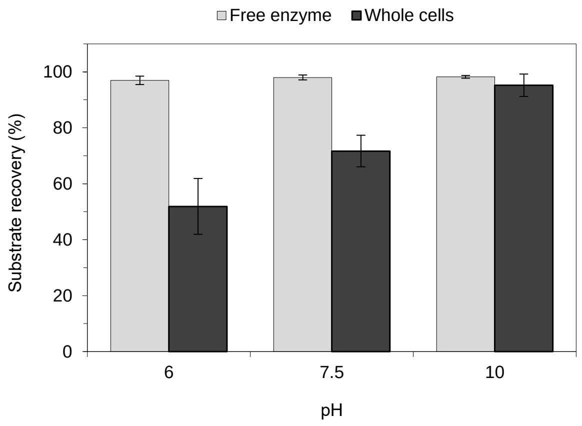

Supplementary Fig. 3. Extracted substrate 1*E/Z* from the biotransformations with free enzyme and whole cells at different pH. Biotransformations were performed with 10 mg of whole cells or 1 mg/mL of partially purified enzyme in 1 mL of 10 mM **1*E/Z***, 1% DMSO, 0.2% SDS, and 20 mM citric acid buffer at pH 6.0 at 30 ºC for 24 h. The bar chart represents the average of technical duplicates. Data are presented as mean values +/- SD.

Methacrylate microbeads functionalized with epoxy (EP403/S), epoxy-butyl (ECR8285), and amino-epoxy (HFA403) groups (Supplementary Fig. 4A-B) were employed for enzyme immobilization. 1 mg/g of protein could be immobilized on ECR8285 and HFA403, while 0.76 mg/g were immobilized on EP403/S (Supplementary Fig. 4C). However, no enzyme activity was recovered after immobilization on ECR8285. Considering the substrate/product sticking issues observed with EP403 microbeads before, HFA403 was selected as support for *Aac*SHC immobilization.

A

B

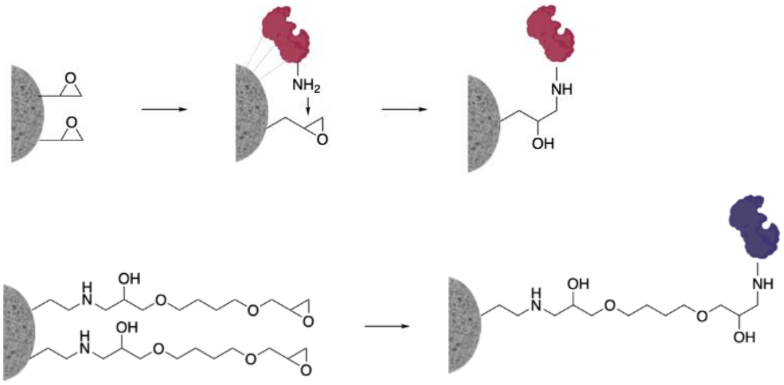

C

Support	Functional group	Pore diameter (nm)	Particle size (µm)	Immobilization yield (%)	Recovered activity (%)
EP403/S	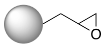	40-60	100-300	75.6	>99
ECR8285	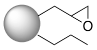	50-70	150-300	>99	<1
HFA403	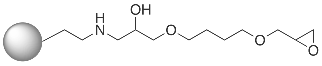	40-60	100-300	>99	42.5

Supplementary Fig. 4. *Aac*SHC immobilization on methacrylate microbeads. A) Scheme of the enzyme immobilization on EP403/S and ECR8285. Firstly, the protein (in red) is hydrophobically adsorbed due to a high ionic strength immobilization buffer. Then, a covalent linkage takes place between the nucleophilic groups (amino, hydroxyl and thiol) on the surface of the enzyme and the epoxy groups on the support. B) Scheme of the enzyme immobilization on HFA403. Firstly, the protein (in purple) is ionically bound to the amino groups on the support and then the covalent bond with the epoxy groups happens. C) Immobilization results. The offered protein loading was 1 mg/g.

The protein loading was increased up to 5 mg/g maintaining the same immobilization efficiency (Supplementary Fig. 5A). In addition, we investigated the use of different molecules to block the remaining epoxy groups on the microbeads after enzyme immobilization, thus providing different microenvironments to the immobilized enzyme. Glycine (more hydrophilic environment) and ethylamine (more hydrophobic environment) are frequently used for this purpose and were tested in this work.[2,3] Both the enzymatic activity and the reusability of the immobilized enzyme showed better results when using ethylamine (Supplementary Fig. 5B-C).

A

Protein loading (mg/g)	Immobilization yield (%)
1	>99
5	>99
10	50

B

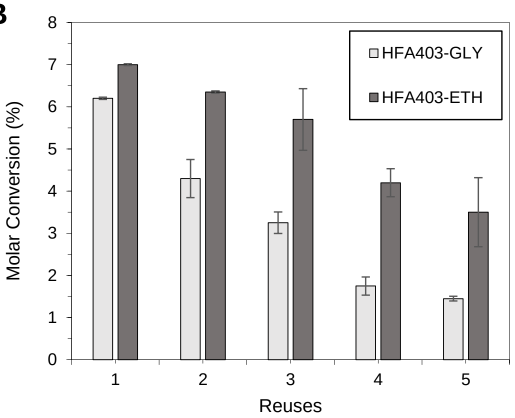

C

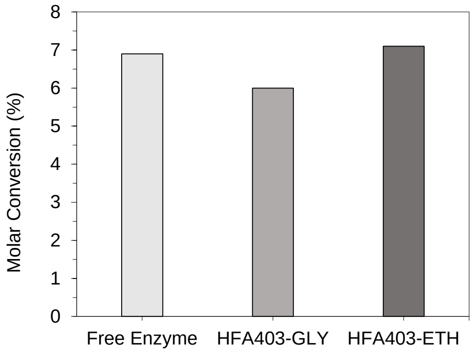

Supplementary Fig. 5. Immobilized *Aac*SHC on HFA403 methacrylate microbeads. A) Protein loading on the immobilization support. B) Effect of the blocking agents, glycine (GLY) and ethylamine (ETH), on the molar conversion referring to the recovered activity after immobilization compared with the free enzyme. The bar chart represents the average of technical duplicates. Data are presented as mean values +/- SD. C) Reusability and stability of the immobilized *Aac*SHC. Each reaction cycle corresponds to 24h. After the reaction, an extraction with ethyl acetate:cyclohexane (1:1) was performed for GC analysis, the immobilized enzymes was washed with buffer and reuse for another reuse. Reactions were performed in technical duplicates.

Supplementary Table 3. Continuous flow biotransformations with immobilized enzyme on methacrylate (HFA403) microbeads. Substrate solution: 10 mM geranyl acetone **1*E/Z***, 1% DMSO, and 10 mM citric acid buffer at pH 6.0. Reactor volume: 1.2 mL. Flow rate: 20-40 µL/min. SD <1%. For the biphasic reactions, the substrate solution (20 mM **1*E/Z***, 1% DMSO, 20 mM citric acid buffer pH 6.0) was mixed in a T-tube with the organic phase (1:1 ethyl acetate and cyclohexane). In case of recirculation, the collected solution with unreacted substrate was used to feed the flow reactor during 6 cycles increasing the overall contact time to 3 h (30 min R.T.) and 6 h (60 min R.T.). The conversion was calculated as the average of at least 3 column volumes. After the reaction was performed, the activity of the immobilized enzyme was tested in batch to confirm that the biphasic system had no influence on the enzyme activity.

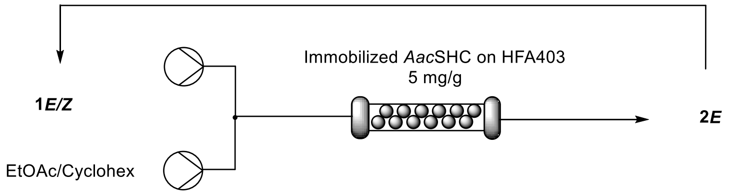

Residence time (min)	Temperature (ºC)	Biphasic system	Recirculation	Addition of surfactant	Molar conversion (%)
30	30	No	No	No	n.d.
		No	No	0.5% Triton	4
		No	Yes	No	4
		Yes	No	No	4
60	50	No	Yes	No	8

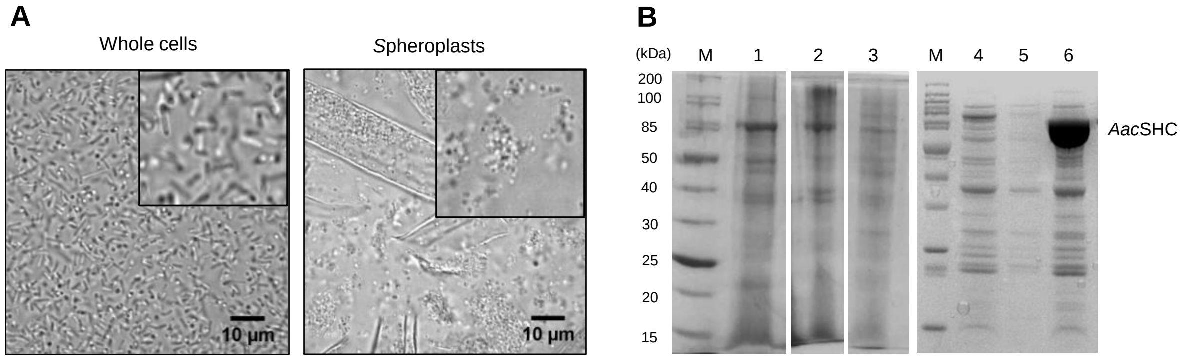

Supplementary Fig. 6. Analysis of *Aac*SHC whole cells and *Aac*SHC spheroplasts. A) Optical microscopy analysis showing the change of cellular shape from whole cells to the typical, more circular, spheroplasts. B) SDS-PAGE analysis of the *Aac*SHC expression and the fractions obtained after the preparation of spheroplasts. Line 1: whole cells with *Aac*SHC. Line 2: spheroplasts corresponding to the pellet after lysozyme/EDTA treatment of whole cells and centrifugation. Line 3: supernatant after lysozyme/EDTA treatment of whole cells and centrifugation. Broad range protein marker from NEB. Line 4: negative control of protein expression after the cells were transformed with the empty pET22b(+) plasmid. Line 5: diluted sample from line 4. Line 6: Overexpression of *Aac*SHC in *E. coli* cells. Source data (scan of uncropped gels) are provided as a Source Data file.

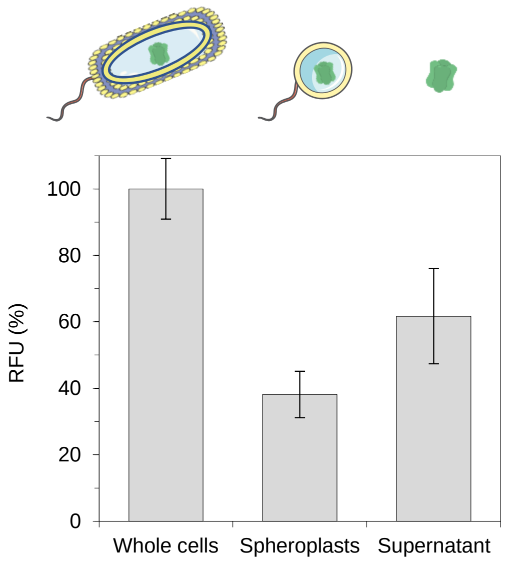

Supplementary Fig. 7. Fluorescence analysis of the content of a cytoplasmatic protein (GFP) in different cellular fractions. Relative fluorescence units (RFU) corresponding to the average of triplicates are shown. Green fluorescent protein (GFP) fluorescence was analysed (λ_{ex}: 480nm; λ_{em}: 512nm) using a Synergy H1 reader. GFP fluorescence of the supernatant after the EDTA-lysozyme treatment is depicted. The buffer solution was used as a blank. Whole cells and spheroplasts producing SHC were also analysed as control (data not shown). On top of the graph, a schematic representation of the GFP (green) in the different preparations is depicted. Graphical representations contain modified Servier Medical Art images (smart.servier.com). The bar chart represents the average of technical duplicates. Data are presented as mean values +/- SD.

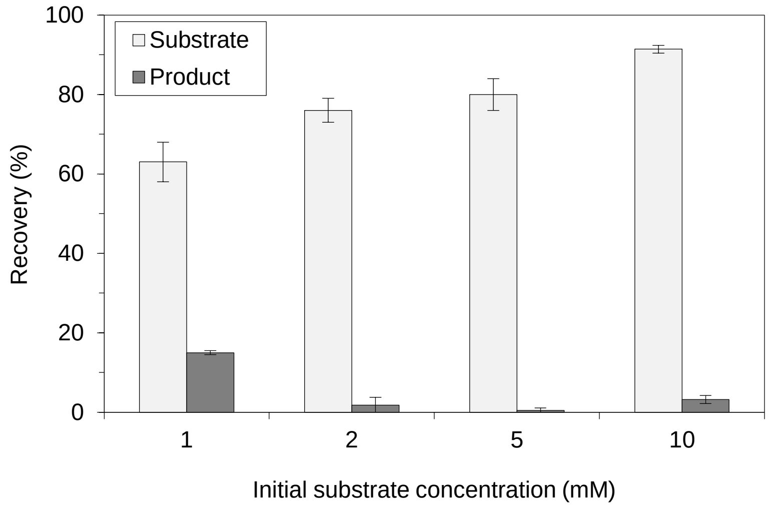

Supplementary Fig. 8. Biotransformations using spheroplast biocatalysts and 1*Z* as substrate. 10 mg of spheroplasts (enzyme variant G600T/L607A/Y420F/A306V) were added in 1 mL of 1-10 mM **1*Z***, 1% DMSO, 0.2% SDS, and 20 mM citric acid buffer at pH 6.0. The percentages of substrate and product were calculated according to a calibration curve analysed by GC. The bar chart represents the average of technical duplicates. Data are presented as mean values +/- SD.

Supplementary Table 4. Effect of the addition of cyclodextrins (CD) and SDS on the biotransformations with spheroplasts in batch mode using 1*E/Z* as substrate. 10 mg of spheroplasts were added in 1 mL of 2 mM geranyl acetone, and 20 mM citric buffer pH 6.0, in presence (2 mM, +CD) or absence (0 mM, -CD) of cyclodextrins, and in presence (0.2%, +SDS) or absence of SDS (0%, -SDS). Reactions were performed in technical duplicates.

Additive	Substrate recovery (%)	Product recovery (%)
+ CD	57	42
- CD	56	47
+ SDS	55	45
- SDS	56	41

Supplementary Table 5. Influence of the lyophilization of spheroplasts on the performance of the biocatalysts at different reaction scale. 10 mg of spheroplasts were added in 1 mL of 2-5 mM geranyl acetone **1*E/Z***, 1% DMSO, 0.2% SDS, and 20 mM citric acid buffer at pH 6.0. Reactions were performed in technical duplicates.

1*E/Z* concentration	Molar conversion (%)	
	Before lyophilization	After lyophilization
2 mM	42	41
5 mM	20	23

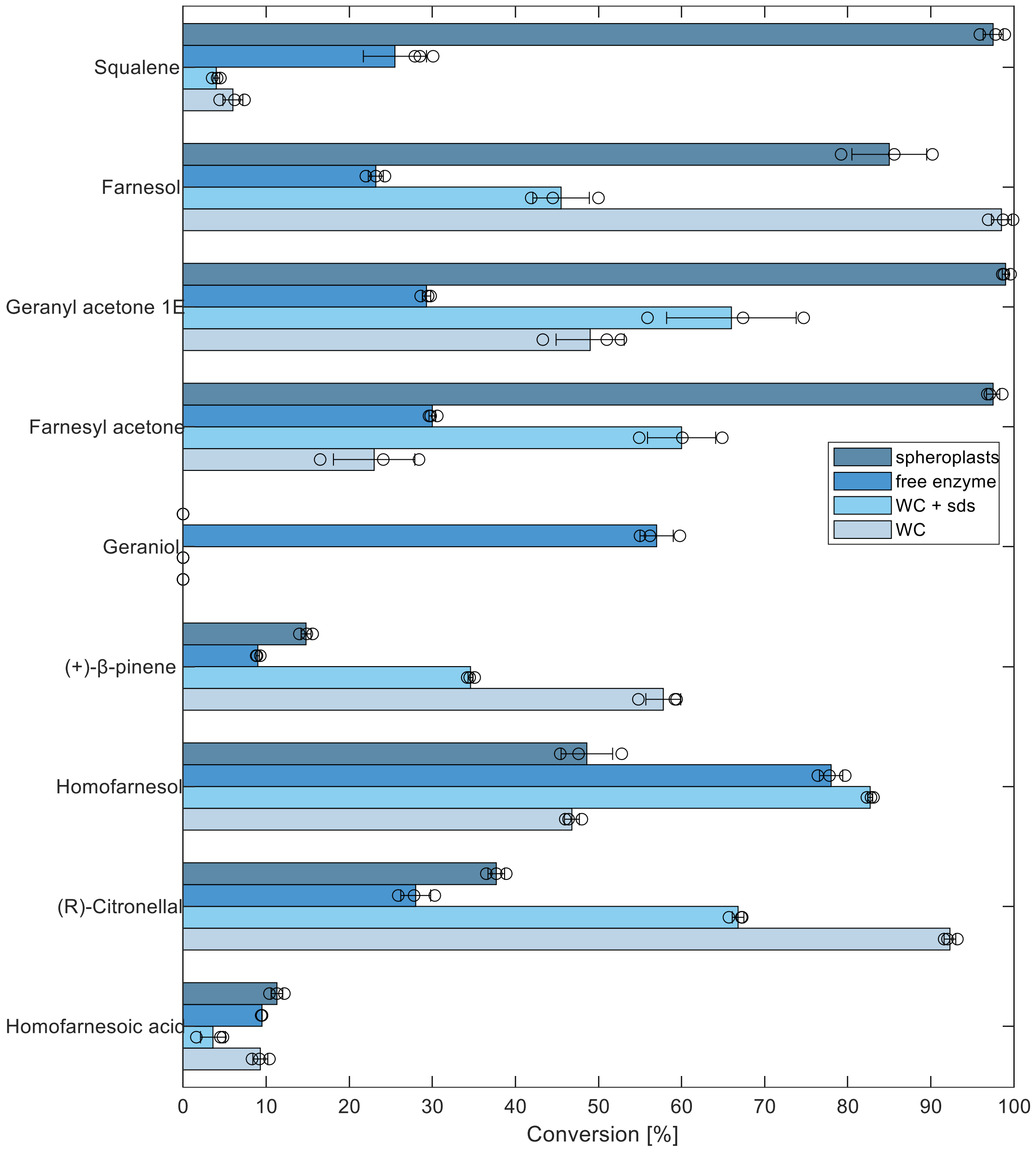

Supplementary Fig. 9. Molar conversion (%) of the results depicted in Fig. 2. Squalene-hopene catalyzed cyclization of the substrates and pure isomers **3, 5, 1E, 7, 9, 11, 13, 15** and **17** employing the WT enzyme and the variants G600F, F365C or A419G Y420C G600A as whole cells (WC), whole cells treated with SDS, cell-free enzyme or spheroplasts. Data are presented as mean values +/- SD. Reaction conditions: 40 gCWW/L whole cells or spheroplasts, 2 mM substrate, shaken at 30 °C for 16 h (biotransformations with substrate **5** were performed at 50 ºC, and with substrates **3** and **9** at 60 ºC). Note that the amount of protein contained in each biocatalyst preparation was: spheroplasts (0.7±0.3 mg/mL), free enzyme (1.3±0.3 mg/mL), WC + SDS (6.5±1.6 mg/mL), WC (5.5±2.7 mg/mL). The free enzyme was used in 0.2 % CHAPS as a membrane mimic. The bar chart represents the average of technical triplicates (dot plots). Data are presented as mean values +/- SD. Quantification was made using dodecane as internal standard. *The protocol for this substrate is different and follows what previously reported by Hammer *et al.*[4]

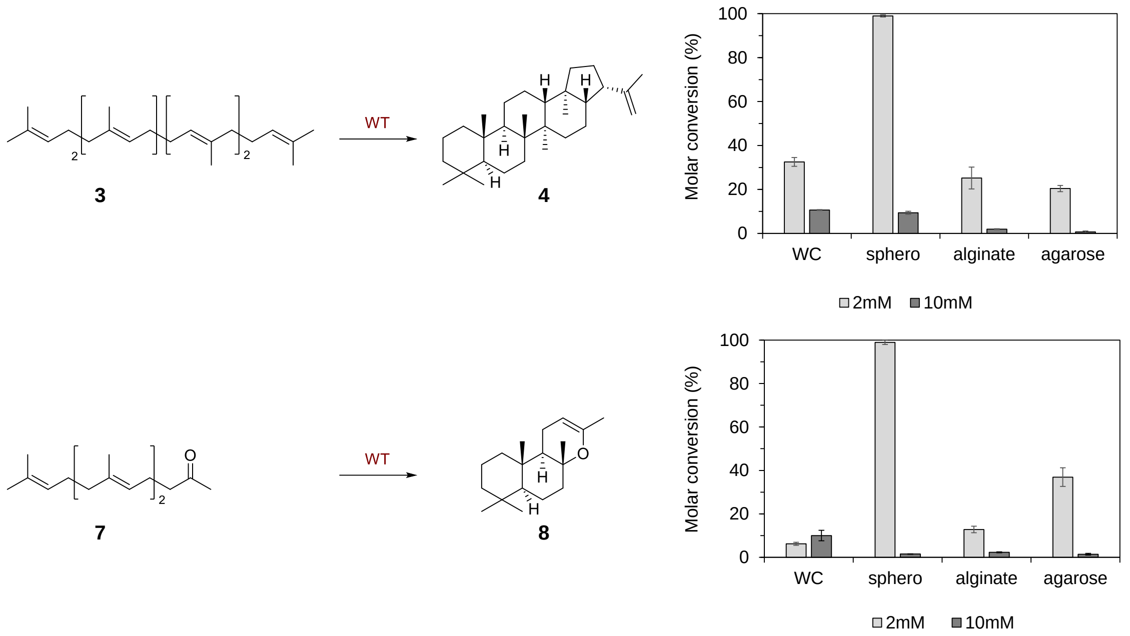

Supplementary Fig. 10. Biotransformations of squalene 3 and *E*,*E*-farnesyl acetone 7 using whole cells (WC), spheroplasts, alginate entrapped spheroplasts or agarose entrapped spheroplasts with 2 (blue bar) or 10 mM (orange bar) substrate. The reactions were shaken at 60 °C for squalene 3 and 30 °C for *E,E*-farnesyl acetone **7** for 16 h. The bar chart represents the average of technical duplicates. Data are presented as mean values +/- SD.

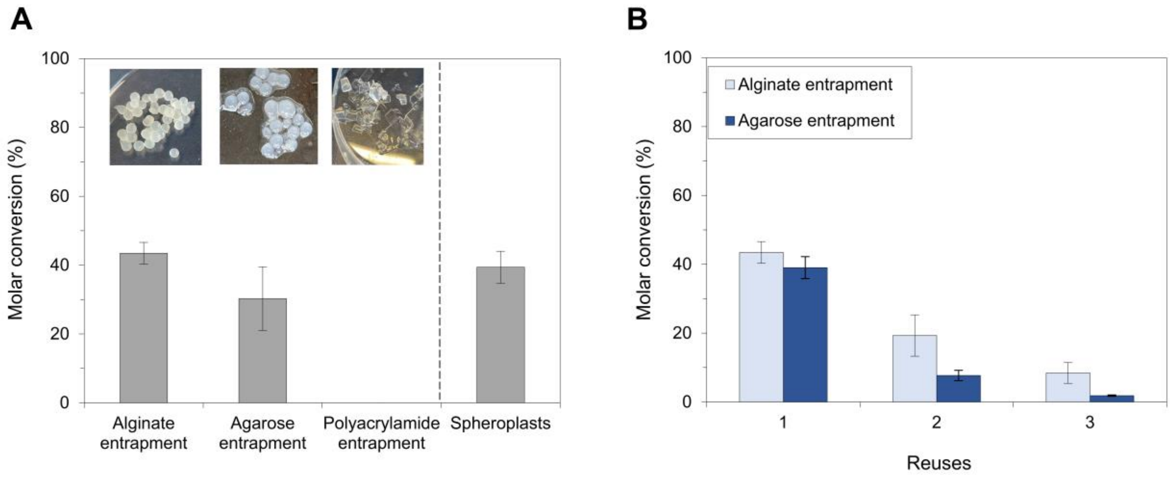

Supplementary Fig. 11. Biotransformations in batch mode with entrapped spheroplasts using 1*E/Z* as substrate. A) 200 mg of entrapped spheroplasts were added to 1 mL of 2 mM geranyl acetone **1*E/Z***, 2 mM cyclodextrin, 0.2% SDS and 20 mM citric acid buffer at pH 6.0. The bar chart represents the average of technical duplicates. Data are presented as mean values +/- SD. Images at the top shows the appearance of the entrapped spheroplasts (size: 1-4 mm). B) Reuses of the entrapped spheroplasts for consecutive biotransformations. Each reuse corresponds to 24 h at 30ºC. After each reuse, the reaction mix was filtered, and the reaction media was replaced by fresh substrate solution. The bar chart represents the average of technical duplicates. Data are presented as mean values +/- SD.

Supplementary Table 6. Continuous flow biotransformations with spheroplasts entrapped into alginate beads using 1*E/Z* as substrate. Substrate solution: 2 mM geranyl acetone **1*E/Z***, 1% DMSO, 0.2% SDS, 2 mM cyclodextrins (CD) and 20 mM citric acid buffer at pH 6.0. Reactor volume: 3.4 mL. Temperature: 30ºC. Flow rate: 50-100 µL/min. SD <1%. In case of recirculation, the collected solution with unreacted substrate was used to feed the flow reactor increasing the overall contact time to 2 h (60 min R.T.).

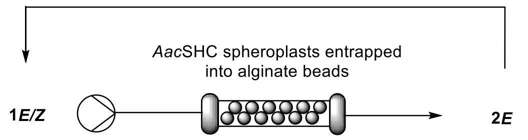

Residence time (min)	Recirculation	Molar conversion (%)
30	No	n.d.
60	Yes	n.d.

Supplementary Table 7. In-line coupled PBR (packed-bed reactors) for the continuous flow biotransformations of the substrate 1*E/Z* with spheroplasts entrapped into alginate beads. Substrate solution: 2 mM geranyl acetone **1*E/Z***, 1% DMSO, 0.2% SDS, 2 mM cyclodextrins (CD) and 20 mM citric acid buffer at pH 6.0. Reactor volume: 3.4 mL. Temperature: 30ºC. Flow rate: 100 µL/min. SD <1%. The residence time (R.T.) of each reactor was 30 min. The contact time refers to the total time that the biocatalyst is in contact with the reaction media.

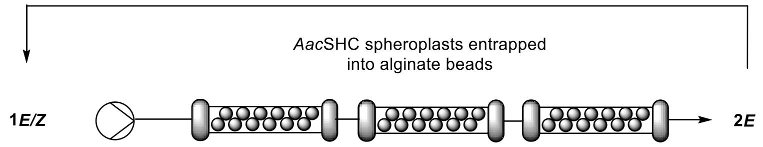

Nº of PBRs in line	Total contact time (min)	Recirculation	Molar conversion (%)
1	30	No	n.d.
2	60	No	n.d.
3	90	No	n.d.
	180	Yes	n.d.
	270	Yes	n.d.
	360	Yes	n.d.
	450	Yes	n.d.

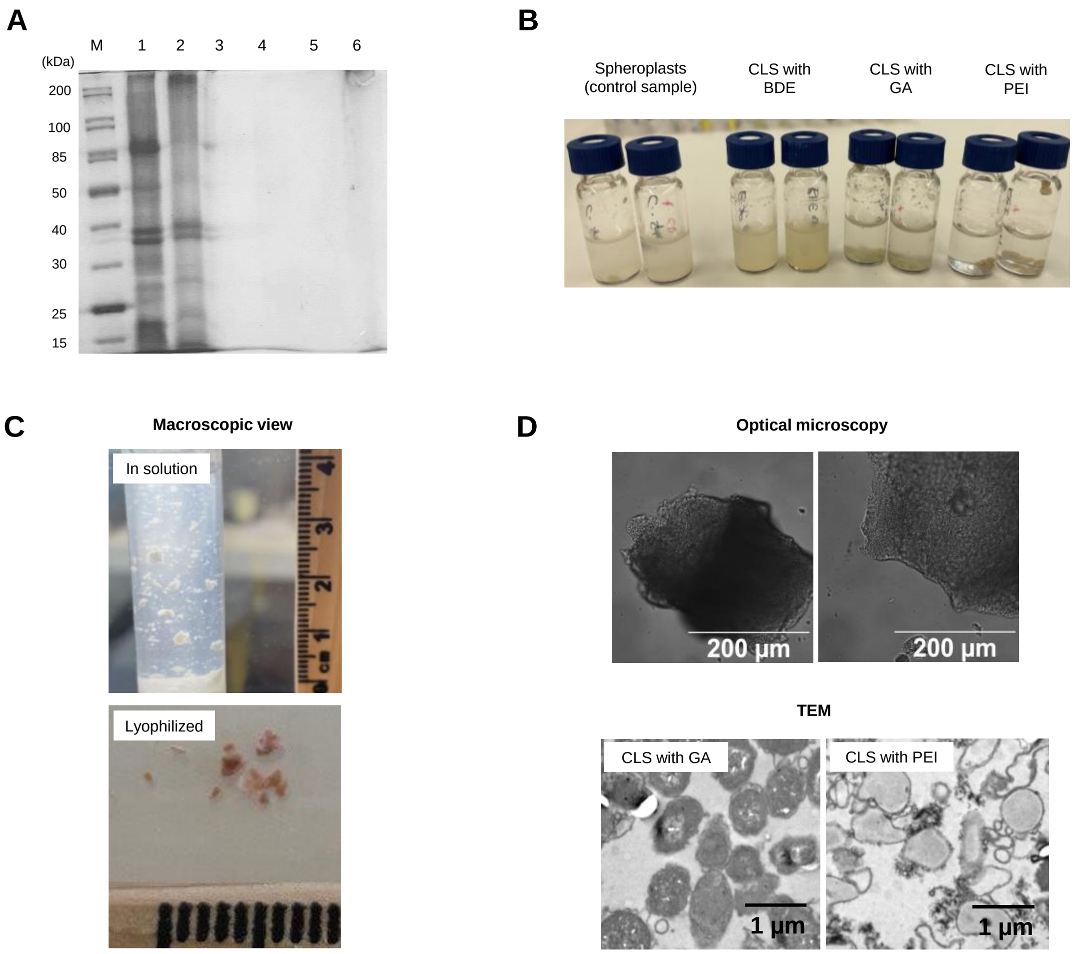

Supplementary Fig. 12. Analysis of the crosslinked spheroplasts (CLS). A) SDS-PAGE. Line 1: flow-through after filtration of spheroplasts (control sample). Line 2: flow-through after filtration of spheroplasts incubated with BDE. Line 3: flow-through after filtration of spheroplasts incubated with GA. Line 4: flow-through after filtration of spheroplasts incubated with PEI. Line 5: flow-through of the filtration after reaction with CLS-GA. Line 6: flow-through of the filtration after reaction with CLS-PEI. Broad range protein marker from NEB. **B)** Appearance of the CLS after incubation with the different crosslinkers: BDE (1,4-butanediol diglycidyl ether), GA (glutaraldehyde) and PEI (polyethyleneimine). **C)** Images of lyophilized an in-solution CLS next to a ruler. **D)** Two optical microscopy images showing the size of CLS; and two TEM images of the CLS after crosslinking with glutaraldehyde (GA) or with polyethyleneimine (PEI). Microscopy images were taken using transmission light in a Nikon Ti2 Eclipse microscope. Objective 40x was used.

Supplementary Table 8. Effect of cyclodextrins (CD) on the biotransformations with CLS using 1*E/Z* as substrate. 10 mg of spheroplasts/CLS were added in 1 mL of 2 mM geranyl acetone , and 20 mM citric buffer pH 6.0, in presence (2 mM, +CD) or absence (0 mM, -CD) of cyclodextrins. The reactions were incubated at 30ºC under shaking for 24 h. Reactions were performed in technical duplicates with a standard deviation <10%.

Biocatalyst	Molar conversion (%)	
	+ CD	- CD
Spheroplasts	42	46
CLS with GA	43	36
CLS with PEI	37	40

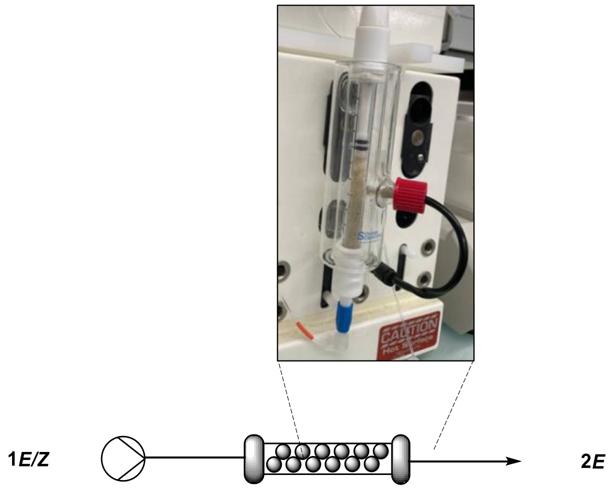

*Aac*SHC crosslinked spheroplasts (CLS)

Residence time (min)	Temperature (ºC)	Molar conversion (%)
60	30	2

Supplementary Fig. 13. CLS with PEI in the flow reactor using 1*E/Z* as substrate. Substrate solution: 2 mM geranyl acetone **1*E/Z*** and 1% DMSO in 20 mM citric acid buffer at pH 6.0. Flow rate: 60 µL/min. The conversion was calculated as the average of at least 3 column volumes.

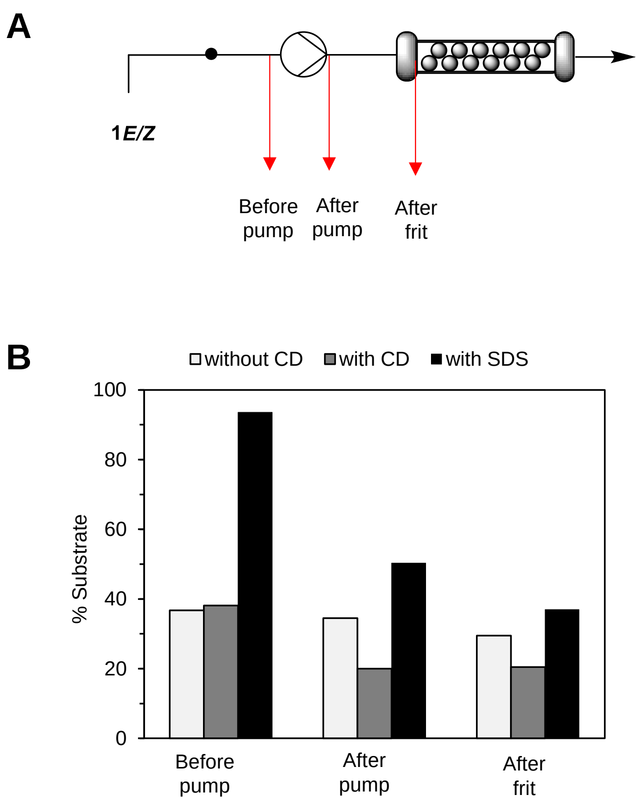

Supplementary Fig. 14. Testing the stickiness of geranyl acetone 1*E/Z* to the tubing of the flow reactor. A) Scheme of the sampling points (represented by red arrows) along the tubing of the flow system. B) Substrate recovered at different points along the tubing of the flow system. Substrate solution: 2 mM geranyl acetone **1*E/Z*** and 1% DMSO in 20 mM citric acid buffer at pH 6.0. In case of additives 0.2% SDS or 2 mM cyclodextrins (CD) were added. Flow rate: 60 µL/min.

Supplementary Table 9. Preliminary analysis of the costs comparing the use of whole cells, spheroplasts and CLS as biocatalysts at lab scale to synthesize the products 2*E* and 4. A) Costs of the biocatalyst preparation (10 mg lyophilized). Prices (€/Kg) from Sigma Aldrich (16.08.2022, Germany). CLS: crosslinked spheroplasts. *For the cost of the lysozyme, the price of the larger format commercially available was considered (100 g) and extrapolated to 1 Kg. B) Costs of the reaction set-up. C) Overall costs calculated by considering both the biocatalyst preparation and the reaction set-up. Calculated costs (€)/$mg_{product}$ were obtained from the results depicted in Fig.S9, considering the reusability of CLS (at least 4 reuses) showed in Fig. 4.

A

Biocatalyst	Reagent	Price (€/Kg)	Reagent (mg) in reaction	Cost (€ x10^{-6})	Total cost (€)
Whole cells	-	-	-	-	-
Spheroplasts	EDTA	65.8	0.03	1.9	0.0035
	NaCl	13.7	0.88	12	
	Sucrose	79.7	10	797	
	Lysozyme	27,200*	0.1	2,720	
**Biocatalyst**	**Reagent**	**Price (€/L)**	**Reagent (mL)**	**Cost (€ x10^{-6})**	**Total cost (€)**
CLS	Glutaraldehyde (25%)	108	0.008	864	0.0044

B

Biocatalyst	Additive	Price (€/Kg)	Additive (mg)/$mL_{reaction}$	Cost (€/$mL_{reaction}$)	Total cost (€)
Whole cells	SDS	579	2	0.0012	0.0363
Spheroplasts	-	-	-	-	-
CLS	-	-	-	-	-

C

Product	Biocatalyst	Product yield (mg)/mL reaction	Biocatalyst (mg) to produce 1 $mg_{product}$	Calculated cost (€)/$mg_{product}$	Calculated cost (€) for 2 mg of product
**2*E***	Whole cells (SDS)	0.256	38.9	0.005	0.009
	Spheroplasts	0.385	25.9	0.009	0.018
	CLS	0.385	25.9	0.004	0.004
**4**	Whole cells (SDS)	0.033	304.4	1.105	2.210
	Spheroplasts	0.801	12.5	0.004	0.009
	CLS	0.801	12.5	0.005	0.005

Supplementary Table 10. Summary of all the immobilization strategies followed in this work. Conversions were calculated from a biotransformation in 1 mL using 2-10 mM of geranyl acetone **1*E/Z*** after 24 h at 30ºC. PEI (polyethyleneimine), GA (glutaraldehyde), BDE (1,4-butanediol diglycidyl ether). n.t. (not tested).

Biocatalyst	Type of immobilization	Immobilization yield [a] [b] (%)	Retained activities [c] (%)
Free enzyme	Methacrylate beads Ep-EP403/S	76	>99
	Methacrylate beads ECR8285	>99	<1
	Methacrylate beads HFA403	>99	43
Whole cells	Methacrylate beads Gx-EP403/S	85	n.t.
	Alginate entrapment	>80	>99
Spheroplasts	Alginate entrapment	>80	>99
	Agarose entrapment	n.t.	77
	Polyacrylamide entrapment	n.t.	<1
	PEI crosslinking	>90	>99
	GA crosslinking	>90	>99
	BDE crosslinking	<10	n.t.

[a] Immobilization yield of the free enzyme was calculated following equation 1:

$$(1) \quad \frac{\text{protein in the initial solution- protein in the supernatant after immobilization}}{\text{protein in the initial solution}} \times 100$$

[b] Immobilization yield for whole cells and spheroplasts was calculated following equation 2:

$$(2) \quad \frac{\text{OD}_{600} \text{ of the initial solution - OD}_{600} \text{ of the supernatant after immobilization}}{\text{OD}_{600} \text{ of the initial solution}} \times 100$$

[c] Retained activities were calculated following equation 3:

$$(3) \quad \frac{\text{conversion with the immobilized preparation (\%)}}{\text{conversion with the corresponding free preparation (free enzymes, whole cells or spheroplasts) (\%)}} \times 100$$

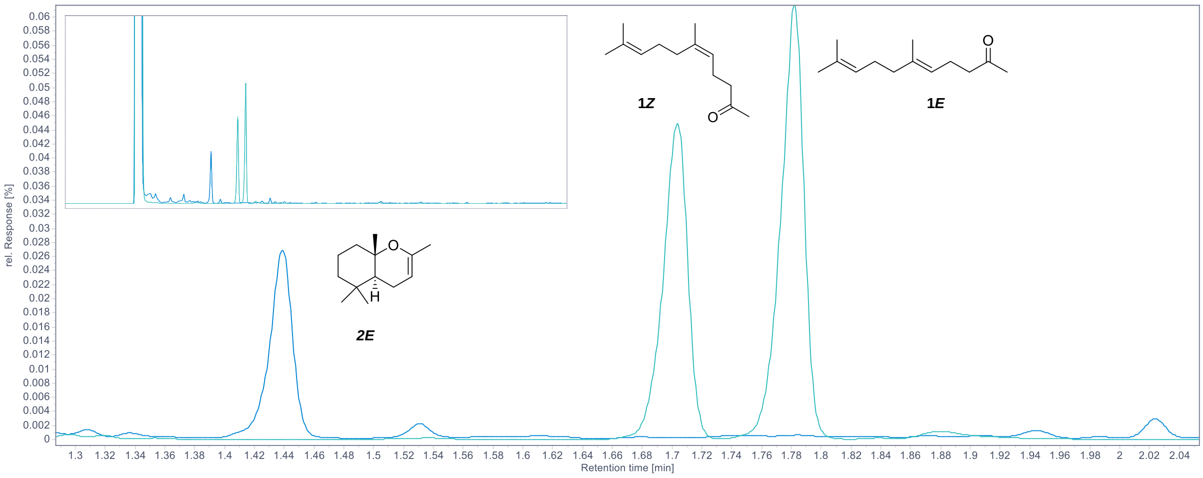

Supplementary Fig. 15. Gas chromatogram of substrate: geranyl acetone **1*E/Z*** (**1*E*:** 1.78 min; **1*Z***: 1.70 min) and product **2*E*** (1.44 min).

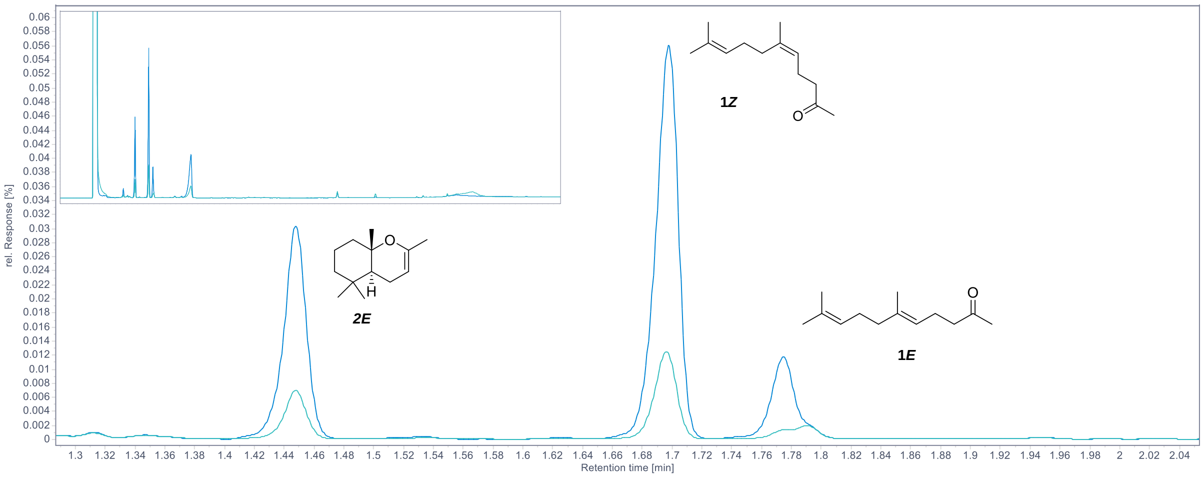

Supplementary Fig. 16. Gas chromatogram of biotransformations: with whole cells and with spheroplasts.

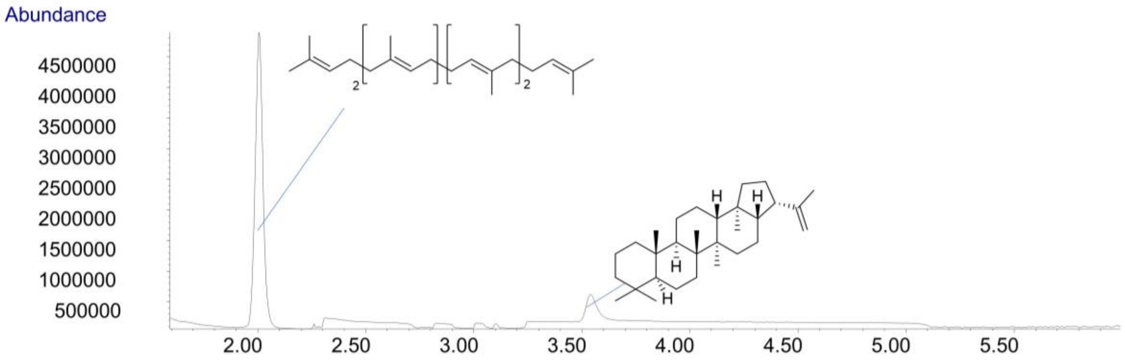

Supplementary Fig. 17. Gas chromatogram of squalene **3** to hopene **4.**

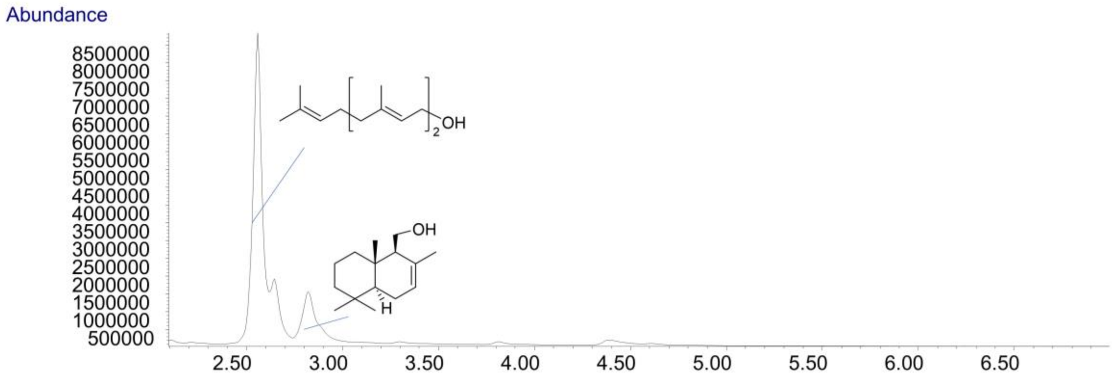

Supplementary Fig. 18. Gas chromatogram of *E,E*-farnesol **5** to drimenol **6.**

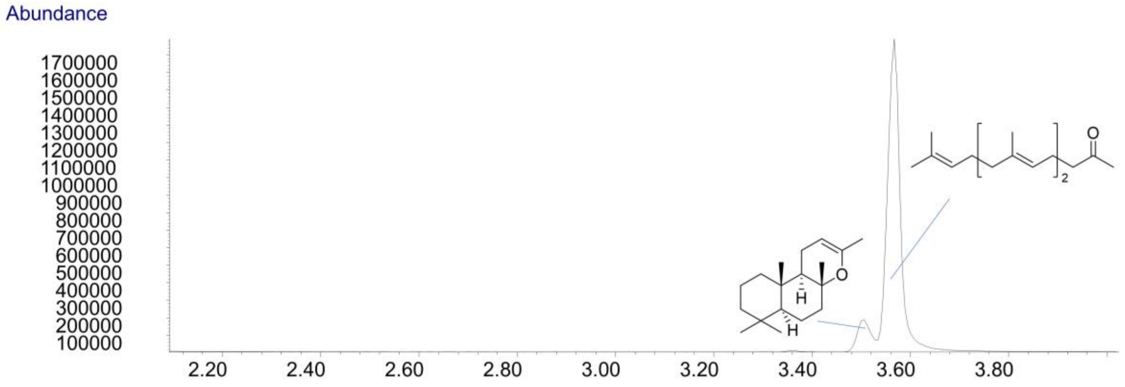

Supplementary Fig. 19. Gas chromatogram of *E,E*-farnesyl acetone **7** to sclareoloxide **8.**

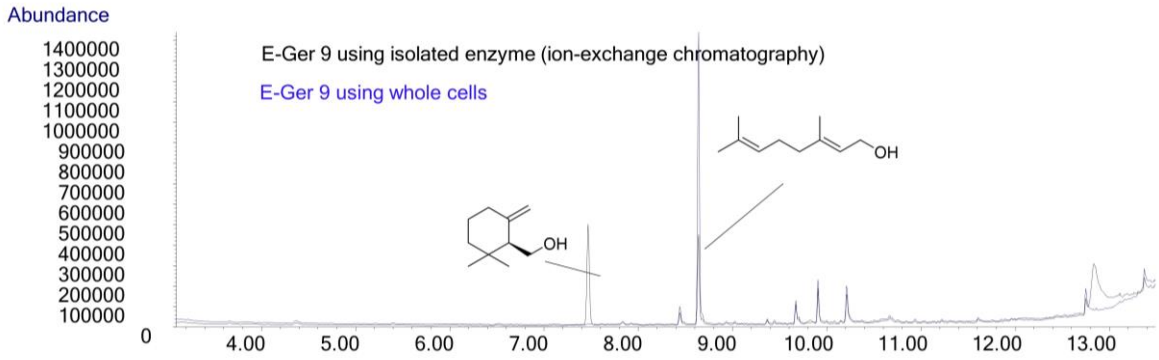

Supplementary Fig. 20. Gas chromatogram of *E*-Geraniol **9** to γ-cyclogeraniol **10.**

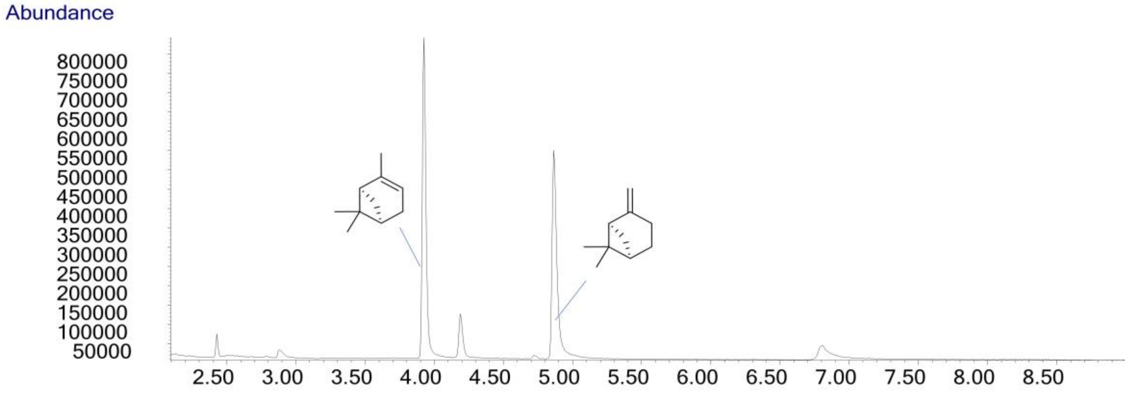

Supplementary Fig. 21. Gas chromatogram of (+)-β-Pinene **11** to (+)-α-Pinene **12.**

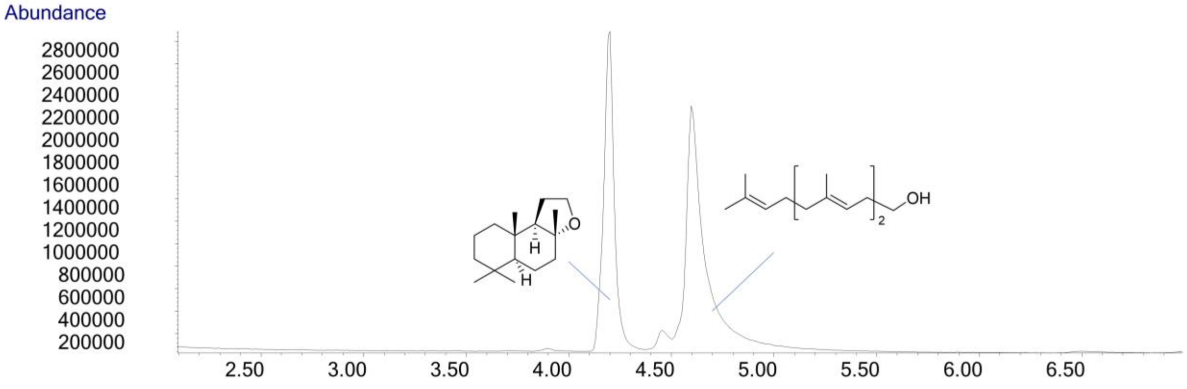

Supplementary Fig. 22. Gas chromatogram of Homofarnesol **13** to (–)-ambroxide **14.**

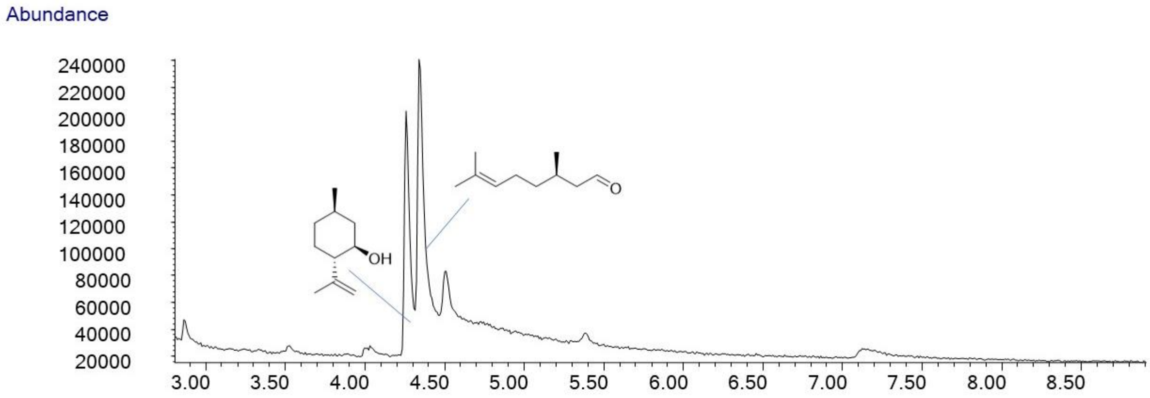

Supplementary Fig. 23. Gas chromatogram of (R)-Citronellal **15** to (–)-Isopulegol **16.**

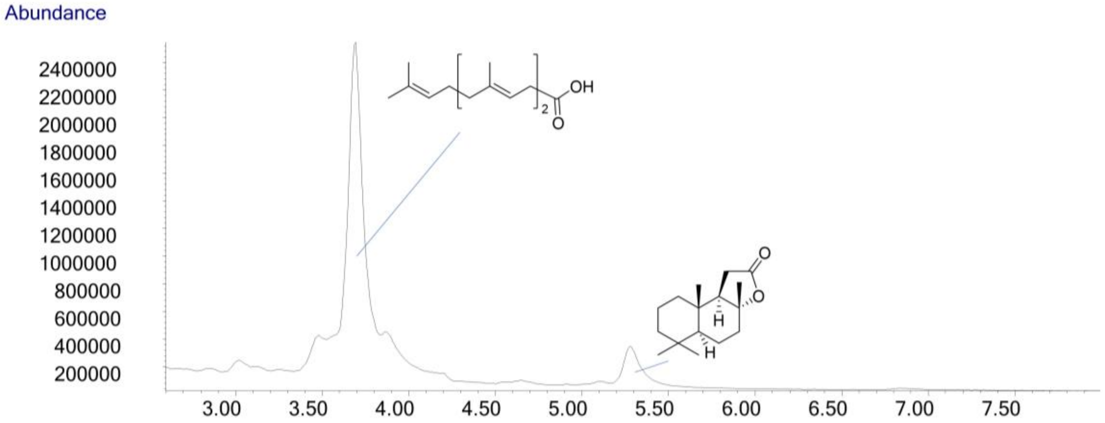

Supplementary Fig. 24. Gas chromatogram of Homofarnesoic acid **17** to sclareolide **18.**

2. Supplementary Methods

Activity assessment of purified *Aac*SHC. The activity of *Aac*SHC (0.02 mg/mL or 0.1 mg/mL of free enzyme; 20 mg of immobilized enzyme) was assessed by monitoring the cyclization of 10 mM geranyl acetone in citric acid buffer (10 mM, pH 6.0) in a total reaction volume of 1 mL. 1 % DMSO was used as co-solvent. The reactions were incubated at 30 °C for 20 h at 300 rpm. The samples were extracted with 1 mL of 1:1 ethyl acetate:cyclohexane. After centrifugation (12,000 rpm, 10 min at 20 °C), the solvent phase was analyzed by GC-FID.

Immobilization of *Aac*SHC on commercially available methacrylate supports.

- *Immobilization on EP403 and ECR8285*: 1 g of the support was suspended in 10 mL of the enzyme solution in 1M sodium phosphate buffer, pH 7.0. The suspension was incubated overnight under orbital shaking at room temperature. Afterwards, the support was filtered and washed with an excess of distilled water. Any unreacted epoxy groups were blocked by an overnight incubation step with 4 mL of blocking buffer (3 M glycine).[5] The blocking step was carried out under orbital shaking at room temperature. Finally, the immobilized enzyme was filtered, washed with an excess of distilled water, and stored at 4 °C.
- *Immobilization on HFA403*: 1 g of the support was suspended in 10 mL of the enzyme solution in 5 mM sodium phosphate buffer, pH 7.0. The suspension was incubated overnight under orbital shaking at room temperature. Afterwards, the support was filtered and washed with an excess of distilled water. Any unreacted epoxy groups were blocked by an overnight incubation step with 4 mL of blocking buffer (3 M glycine).[6] The blocking step was carried out under orbital shaking at room temperature. Finally, the immobilized biocatalyst was filtered, washed with an excess of distilled water, and stored at 4 °C. Given that improved activity and stability was observed when glycine was replaced with ethylamine, the quenching of unreacted epoxy groups on the carrier surface was performed with 5% ethylamine for all immobilized preparations used under flow conditions.

Modification of the immobilized biocatalyst microenvironment. Modification of the immobilized biocatalyst microenvironment can be achieved by quenching the unreacted epoxy groups on the carrier surface using different blocking agents. The most promising immobilized preparation, *Aac*SHC immobilized on HFA403, was therefore treated with blocking buffer containing different blocking agents, including 3 M glycine and 5 % ethylamine.[2]

Immobilization of cells/spheroplasts by entrapment.

- *Alginate entrapment:* 1.8 mL of 4% alginate was prepared in 20 mM citric buffer pH 6.0. 50 mg of cells/spheroplasts resuspended in 0.2 mL of 20 mM citric buffer pH 6.0 were added to the alginate solution. Then, the alginate mixture was poured dropwise using a syringe with a needle on a petri dish containing a solution of 200 mM $CaCl_2$. The resulting alginate beads were incubated for at least 2 hours in the $CaCl_2$ solution. Finally, the alginate beads were washed with 20 mM citric buffer pH 6.0.
- *Agarose entrapment:* 4% of agarose was dissolved in 20 mM citric buffer pH 6.0 by heating. A 50 mL beaker containing sunflower oil was placed into ice with gentle magnetic stirring. Once the agarose solution was below 50ºC, 50 mg of spheroplasts resuspended into 1 mL of buffer were added. Then, the mixture was poured dropwise into the beaker containing sunflower oil to entrap the spheroplasts into agarose beads. Finally, the oil was removed by washing with hexane and then with 20 mM citric buffer pH 6.0.
- *Polyacrylamide entrapment*: 50 mg of spheroplasts were added to a solution containing 1 mL of 40% acrylamide, 0.5 mL of 20 mM citric buffer pH 6.0, 22.5 µL of 10% APS, and 7.5 µL of TEMED. The hydrogel was poured on a petri dish as a layer. After polymerization, the polyacrylamide hydrogel was cut into small pieces for a better handling washed with 20 mM citric buffer pH 6.0.

Thermolysis purification.[7] Spheroplasts or lyophilized cells were resuspended in 1 mL Lysis buffer (200 mM citrate, 0.1 % EDTA, pH 6.0) and incubated for 60 min at 70 °C. The cell suspension was centrifuged (14000 x g, 1 min) and the supernatant was kept for analysis. As the enzyme is membrane-bound, 1 mL 1%-CHAPS buffer (100 mM citrate, pH 6.0) was added to extract it from the cell pellet by shaking at room temperature for 1d, 600 rpm. After subsequent centrifugation (14000 x g, 1 min) the supernatant containing the *Aac*SHC was transferred to a new tube followed by SDS-PAGE analysis and determination of enzyme concentration by using the Nanodrop 1000 (Agilent, Santa Clara, US). Therefore the "Protein A280" mode was chosen with MW= 71439 Da and molar extinction coefficient ε = 185180 as protein specific data calculated using the online-software ProtParam.[8]Ion-exchange chromatography purification. 1 g_{CWW} cells were resuspended in lysis buffer (3 ml per gram cells, 200 mM citrate, 1 mM EDTA, pH 6.0) and homogenized using a tissue homogenizer. After addition of phenylmeth-anesulfonylfluoride (1 mM) and DNAse (~10 µg/ml), the resulting suspension was disrupted by ultrasonic treatment (Branson Sonifier 250, duty cycle 35%, output control 4, 4 min). After centrifugation (38.700 x g, 45 min, 4 °C), the pellet was resuspended in solubilization buffer (1 ml per gram pellet, 1% CHAPS, 60 mM citrate, pH 6.0) and incubated overnight by gently mixing at 4 °C. Triton X-100 or CHAPS (3-[(3-cholamidopropyl) dimethylammonio]-1-propanesulfonate) were used as detergents. After centrifugation (38.700 x g, 45 min, 4 °C) the pellet was discarded, the solution was subsequently heat shocked at 50 °C for 15 min, and precipitated *E. coli* proteins were removed via centrifugation (38.700 x g, 45 min, 4 °C). To obtain SHC solutions with higher purity, the CHAPS–solubilized SHC variants were further purified by diluting with water (1:5, Milli-Q water) and loading onto a column with DEAE-Sepharose (Sigma Aldrich, diethylaminoethyl-sephacel). The bound proteins were washed with equilibration buffer (0.2 % CHAPS, 12 mM citrate, pH 6.0) and eluted with elution buffer (0.2 % detergent, 12 mM citrate, 200 mM NaCl, pH 6.0).

Analytical biotransformations in 2 mL GC screw cap-vials.

- *Whole cells*: 10 mg_{CDW} (= 40 mg_{CWW}) cells were resuspended in 990 µL *dd*H_2O. 10 µL of a substrate/DMSO stock (c_{end}, substrate = 2 mM or 10 mM) were added to start the reactions. Reactions were shaken at the respective temperature for 16 h. Reactions were extracted two times by adding 500 µL cyclohexane:ethyl acetate (1:1) and vortexing for 2 min. After centrifugation (4000 x g, 5 min) the organic phases were combined and analyzed via GC/MS/PAL-Sampler and GC-FID. Quantification was made by dodecane as internal standard.
- *Whole cells treated with SDS:* The reactions with SDS-treated cells were performed analogously adding 0.2 % SDS to get a ratio of c_{SDS}:$c_{cells,CWW}$ = 0.05.
- *Spheroplasts:* The reactions with spheroplasts were performed analogously using 40 mg_{CWW}
- *Free enzyme*: After thermolysis purification the extracted AacSHC variants were diluted 1:5 with *dd*H_2O and to 990 µL of the enzyme preparation was added 10 µL of a substrate/DMSO stock ($c_{end,substrate}$ = 2 mM or 10 mM) to start the reaction. The reactions were shaken at respective temperature for 5 h. Reactions were extracted two times by adding 500 µL cyclohexane:ethyl acetate (1:1) and vortexing for 2 min. After centrifugation (4000 x g, 5 min) the organic phases were combined and analyzed via GC/MS/PAL-Sampler and GC-FID. Quantification was made by dodecane as internal standard. For the *E*-geraniol **9** reaction the Ion-exchange chromatography purified enzyme was used.
- *Alginate beads/ Agarose beads:* 200 mg beads were employed, and the reactions were carried out analogously to the whole cell approaches.

Preparation of spheroplasts with GFP and analysis of the cytoplasmic protein. 1 mL of an overnight culture of *E. coli* BL21(DE3) transformed with the plasmid sGFP_pET28b was used to inoculate 50 mL of LB medium containing 30 µg/mL Kanamycin. The culture was incubated at 37ºC, 150 rpm until the OD_{600nm} reached 0.6. Then, 1 mM IPTG was added to induce expression and the cells were grown at 37 ºC for 3 h. Afterwards, the cells were

harvested by centrifugation (4000 g, 20 min, 4 ºC). 100 mg of whole cells where washed (x3) with 20 mM citric acid buffer at pH 6.0. Then, the protocol for spheroplast preparation was applied, saving the supernatant after EDTA-lysozyme treatment for fluorescence measurement. Finally, whole cells and spheroplasts were sonicated for 8 min (5s ON, 5s OFF at 20% amplitude). After centrifugation (17,000 g, 3 min), 100 µL of soluble GFP were transferred to a 96-well plate and the fluorescence was analysed (λ_{ex}: 480nm; λ_{em}: 512nm) using a Synergy H1 reader. The buffer solution was used as a blank for fluorescence measurements. Whole cells and spheroplasts producing *Aac*SHC were also analysed as control.

<u>TEM analysis</u>

Samples were centrifuged after each step with a table centrifuge at 12,000 rpm. After removal of the supernatant bacteria were submerged with fixative witch was prepared as follows: 2.5% glutaraldehyde (Agar Scientific, Stansted, Essex, UK) in 0.15M HEPES (Sigma-Aldrich, Buchs, Switzerland) with an osmolarity of 670 and adjusted to a pH of 7.35. The cells remained in the fixative at 4°C for at least 24h before being further processed. They were then washed with 0.15 M HEPES three times for 5 min, postfixed with 1% OsO4 (Electron Microscopy Sciences, Hatfield, USA) in 0.1 M Na-cacodylate-buffer (Merck, Darmstadt, Germany) at 4°C for 1 h. Thereafter cells were washed in 0.05 M maleic acid (Merck, Darmstadt, Germany), pH 5.0, three times for 5 min and dehydrated in 70, 80, and 96% ethanol (Alcosuisse, Switzerland) for 15 min each at room temperature. Subsequently, cells were immersed in 100% ethanol (Merck, Darmstadt, Germany) three times for 10 min, in acetone (Merck, Darmstadt, Germany) two times for 10 min, and finally in acetone-Epon (1:1) overnight at room temperature. The next day, cells were embedded in Epon (Sigma-Aldrich, Buchs, Switzerland) and left to harden at 60°C for 5 days. 75nm sections were produced with an ultramicrotome UC6 (Leica Microsystems, Vienna, Austria). The sections, mounted on 200 mesh copper grids, were stained with UranyLess (Electron Microscopy Sciences; Hatfield, USA) and lead citrate with an ultrostainer (Leica Microsystems, Vienna, Austria). Sections were then examined with a transmission electron microscope (Tecnai Spirit, FEI, Brno, Czech Republic) equipped with a digital camera (Veleta, Olympus, Soft Imaging System, Münster, Germany).

3. Supplementary References

1. Irvin, R. T., MacAlister, T. J. & Costerton, J. W. Tris(hydroxymethyl)aminomethyl buffer modification of Escherichia coli outer membrane permeability. *J. Bacteriol.* **145**, 1397–1403 (1981).

2. Alsafadi, D. & Paradisi, F. Covalent immobilization of alcohol dehydrogenase (ADH2) from Haloferax volcanii: How to maximize activity and optimize performance of halophilic enzymes. *Mol. Biotechnol.* **56**, 240–247 (2014).

3. Mateo, C. *et al.* One-step purification, covalent immobilization, and additional stabilization of poly-His-tagged proteins using novel heterofunctional chelate-epoxy supports. *Biotechnol. Bioeng.* **76**, 269–276 (2001).

4. Hammer, S. C., Marjanovic, A., Dominicus, J. M., Nestl, B. M. & Hauer, B. Squalene hopene cyclases are protonases for stereoselective Brønsted acid catalysis. *Nat. Chem. Biol.* **11**, 121–126 (2015).

5. Mateo, C. *et al.* Immobilization-stabilization of enzymes by multipoint covalent attachment on supports activated with epoxy groups. in 47–55 (2006). doi:10.1007/978-1-59745-053-9_4.

6. Mateo, C. *et al.* Epoxy-amino groups: A new tool for improved immobilization of proteins by the epoxy method. *Biomacromolecules* **4**, 772–777 (2003).

7. Ren, X., Yu, D., Han, S. & Feng, Y. Thermolysis of recombinant *Escherichia coli* for recovering a thermostable enzyme. *Biochem. Eng. J.* **33**, 94–98 (2007).

8. Gasteiger, E. *et al.* The proteomics protocols handbook - Chapter 52: protein identification and analysis tools on the ExPASy server. in *The Proteomics Protocols Handbook* 571–607 (2005).
