# Supplementary material for: Spheroplasts preparation boosts the catalytic potential of a squalene-hopene cyclase
Source: Nat Commun. 2022 Oct 21;13:6269. doi: 10.1038/s41467-022-34030-0 (PMC9586974; doi:10.1038/s41467-022-34030-0)
Supplement: Supplementary file 4 — Source Data [file 41467_2022_34030_MOESM4_ESM.zip › NCOMMS-22-10216D Source Data.docx]

Uncropped scans of gel presented in the Supplementary Fig. 6B
